# CircAtlas: an integrated resource of one million highly accurate circular RNAs from 1070 vertebrate transcriptomes

**DOI:** 10.1186/s13059-020-02018-y

**Published:** 2020-04-28

**Authors:** Wanying Wu, Peifeng Ji, Fangqing Zhao

**Affiliations:** 1 Computational Genomics Lab, Beijing Institutes of Life Science, Chinese Academy of Sciences, Beijing, 100101 China; 2 University of Chinese Academy of Sciences, Beijing, 100049 China; 3 Center for Excellence in Animal Evolution and Genetics, Chinese Academy of Sciences, Kunming, 650223 China

**Keywords:** circRNA, Functional prioritization, circAtlas, Multiple conservation score

## Abstract

Existing circular RNA (circRNA) databases have become essential for transcriptomics. However, most are unsuitable for mining in-depth information for candidate circRNA prioritization. To address this, we integrate circular transcript collections to develop the circAtlas database based on 1070 RNA-seq samples collected from 19 normal tissues across six vertebrate species. This database contains 1,007,087 highly reliable circRNAs, of which over 81.3% have been assembled into full-length sequences. We profile their expression pattern, conservation, and functional annotation. We describe a novel multiple conservation score, co-expression, and regulatory networks for circRNA annotation and prioritization. CircAtlas can be accessed at http://circatlas.biols.ac.cn/.

## Background

Recent advances in deep sequencing have led to a deeper appreciation of the intricate nature of transcription by facilitating the capture of a milieu of circular RNAs (circRNAs). The development of bioinformatics-based algorithms has promised enhancement in the accuracy and sensitivity of the circRNA identification, along with the determination of internal components and alternative splicing events, from large-scale RNA-sequencing datasets [1]. For example, the CIRI-AS [2] utilizes a spliced junction signature-based algorithm and enables the high-throughput detection of the internal components of the circRNAs. Similarly, the FUCHS [3] predicts the internal structure of the circRNA by mapping to back-splicing junction (BSJ) reads, whereas the CIRCexplorer2 [4] achieves this through the comparison between the poly(A)+ and the poly(A)− RNA-sequencing data. More recently, the CIRI-full [5] was developed for the effective reconstruction of full-length circRNAs and isoform-level quantification, which greatly facilitates the identification of evolutionarily conserved circRNAs.

As a heterogeneous class, the circRNAs have been shown to participate in different aspects of biological processes through diverse and less understood mechanisms. Besides the well-known function of the circRNAs as microRNA sponges, extensive studies have revealed that the circRNAs play important roles in gene regulation, development, and carcinogenesis [6–8]. Recent efforts have shown that certain circular transcripts are involved in innate immune responses, RNA-protein complex formation, and molecular circuitry controlling human pluripotency [9, 10]. A more recent study has revealed that N^6^-methyladenosine (m^6^A) [11] promotes the efficient initiation of protein translation from the circRNAs. Subsequently, Zhou et al. [12] demonstrated that m^6^A was widespread in circRNAs and many m^6^A modification sites in circRNAs were distinct from those in their host genes. In addition, several recent studies have reported that the circRNAs can encode proteins and their translated products may play important roles in various biological processes [13–16]. Although the body of literature on circRNAs has shown a staggering increase in recent years, current knowledge on the circRNA diversity and function remains inadequate.

Given the rapid accumulation of large circRNA datasets and the imperative requirement of delineating their functional importance, various databases (e.g., circBase [17], CIRCpedia v2 [18], circRNADb [19], TSCD [20], CircRiC [21], and MiOncoCirc [7]) have been established to extend the catalog of the annotated circRNAs. Moreover, other interaction databases, such as the starBase [22] and the CircInteractome [23], may provide informative resources that may enable researchers to prioritize the promising candidates. Nevertheless, novel circRNAs far supersede the coverage provided by existing databases, which predominantly focus on the identification of the circRNAs from cell lines or a small collection of tissues (in particular from cancer samples). For example, MiOncoCric, circRic, and TSCD are equipped to detect a set of circRNAs from cancer cell lines or clinical samples, whereas the circRNADb provides information about the circRNAs from human brain and cell lines. Considering that the vast majority of circRNAs are extremely cell-type-specific and usually transcribed at low levels [24], the discovery of novel circRNAs is an ongoing process with continuous improvements. The presence of different species in the circRNA database is essential due to the important role of conservation analysis for gene functional studies. However, the existing circRNA collections have been largely limited to certain well-studied species, such as human and mouse, or represent a combination of very few sequenced species. For example, the circBase was constructed based on the circRNAs from *Homo sapiens*, *Mus musculus*, *Caenorhabditis elegans*, and *Latimeria*. Given the rapid change in circRNA levels during evolution, conservation analysis based on these collections may result in the identification of very few conserved circRNAs by sequence similarity across long evolutionary distances. Furthermore, a systematical and comprehensive annotation of the circRNAs is important for further probing of their functions and associated molecular mechanisms. Nevertheless, the current annotation is far from sufficient and rather incomprehensive, since most of the databases utilize just one or two resources for annotation. For example, the CircNet [25] utilizes only microRNA-binding site prediction for circRNA annotation. Therefore, there is an urgent need for a dedicated database of circRNAs, compiled from closely related species, that provides detailed annotations.

To bridge the large gap between the overwhelming number of circRNAs and their biological functions, we compiled a comprehensive repository of circRNAs, referred to as the circAtlas, from six vertebrates including human, macaque, mouse, rat, pig, and chicken. We integrated 1070 transcriptomes to generate a comprehensive atlas of over one million circRNAs and expression profiles across multiple tissues from these six vertebrates. Conservation of each circRNA across the species, tissues, and individuals was evaluated. Functional annotation of the circRNAs was performed by integrating co-expression profiles with miRNA interaction and RNA-binding protein (RBP) interaction data. Hence, the circAtlas can serve as a comprehensive functional circRNA resource to efficiently browse, annotate, and prioritize the circRNAs and provide insights into their conservation and functions.

## Construction and content

### Construction and functionalities of the circAtlas

**Fig. 1:**
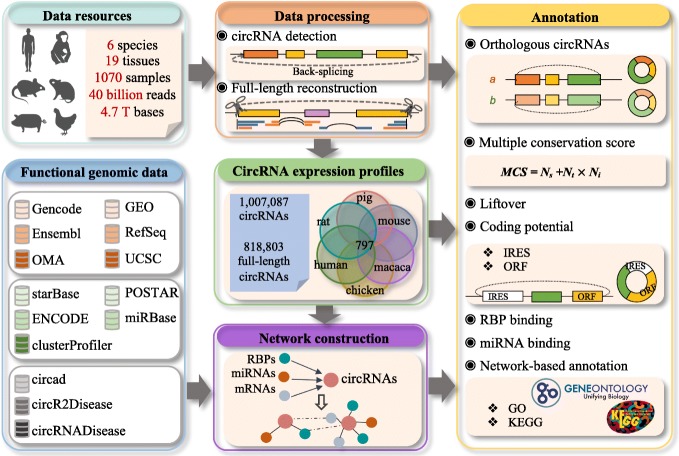
Schematic overview of the construction of circAtlas and associated functionalities

The construction of circAtlas is based on the comprehensive analysis of circRNAs from a compendium of 1070 RNA-seq datasets, encompassing 19 normal tissues collected from six vertebrate species (human, macaque, mouse, rat, pig, and chicken), using current state-of-the-art bioinformatics-based approaches (Additional file 1: Table S1-S3). The content and construction of the circAtlas has been shown in Fig. 1. Briefly, the circRNAs in each species were identified using four detection algorithms, including CIRI2 [26, 27], find_circ [6], CIRCexplorer2 [28], and DCC [29], which have been widely used and extensively tested by previous studies [1, 30–32]. Full-length sequences of the identified circRNAs were then reconstructed using the CIRI-full/CIRI-vis pipeline [5, 33]. Then, the full-length circRNAs were searched for internal ribosome entry sites (IRESs) and ORFs to predict their coding potential. Conservation profiles of the circRNAs were characterized using the multiple conservation score (MCS) scheme, which estimates the conservation of circRNA on three levels, including species, tissues, and individuals. Based on the MCS, an ID assignment scheme was introduced, which included information, including species, conservation, and host gene, for each circRNA. Information on the co-expression network, circRNA-miRNA, and RBP-binding sites were next combined to provide a comprehensive annotation of these circRNAs. The GO and the KEGG databases were used to predict the potential functions for these circRNAs. Meanwhile, the circad, circR2Disease [34], and circRNADisease [26] pipelines were integrated into the circAtlas to evaluate the correlation of circRNAs to various diseases. To facilitate the broad usage of the circAtlas, we have developed a user-friendly, interactive, and open-access web portal for querying and visualizing the circRNAs and their annotations. The portal takes a list or sequences of the circRNAs as an input. If the query circRNAs have been already included in the circAtlas, users can browse and immediately download their sequences, expression profiles, and annotations. Otherwise, the server queries the orthologs of novel circRNAs across species, performs functional annotation, and prioritizes the candidates based on comprehensive annotations. The web application for the circAtlas database was developed using MySQL v.5.6.38 and PHP v.7.0. The entire content of the circAtlas is freely available and can be downloaded from the website (http://circatlas.biols.ac.cn/).

### CircRNA detection and full-length transcript construction

**Fig. 2:**
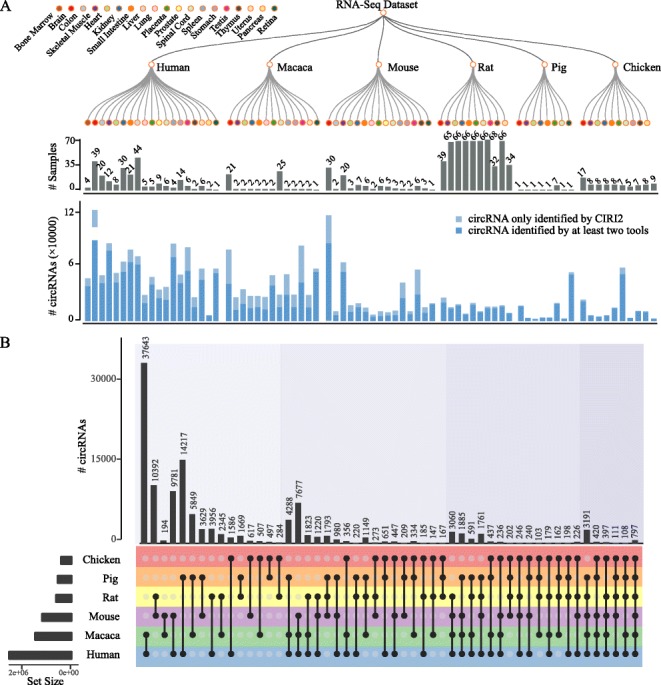
Expression landscape and orthologous circRNAs in six vertebrates. **a** The number of samples and identified circRNAs in each species. The colored circles on the top panel represent different tissues. The plots in gray and blue colors represent the number of samples and identified circRNAs, respectively, in each tissue. **b** An UpSetR visualization of the intersection between sets of orthologous circRNAs. The grid along the bottom has been used to identify intersecting sets (analogous to a Venn diagram). The horizontal bars on the left show the total number of circRNAs detected in each species. The black dots indicate different patterns of sharing between different species, and the vertical bars represent the number of orthologous circRNA pairs for a given pattern

The assembly versions of reference genomes for *Homo sapiens*, *Macaca mulatta*, *Mus musculus*, *Rattus norvegicus*, *Sus scrofa*, and *Gallus gallus* were hg38, rheMac8, mm10, rn6, susScr11, and galGal4, respectively. These were downloaded from GENCODE [35] and Ensembl [36]. For each RNA-seq dataset, four different circRNA detection tools were used, including CIRI2, DCC, CIRCexplorer2, and find_circ, with default parameters. The circRNAs detected by at least two tools and supported by at least two independent BSJ reads were retained for downstream analysis (Fig. 2a). This pipeline was found to generate an average of 167,847 circRNAs for each species. Meanwhile, an adapted CIRI-full pipeline, which utilizes both reverse overlapping information of the circRNAs and the internal landscapes of circRNAs detected by CIRI-AS, was used to reconstruct the full-length circRNA transcripts. Consequently, an average of 136,467 full-length circRNAs for each species was obtained. Finally, we implemented the back-spliced junctions per million mapped fragments (FPM) values as a quantitative proxy of the circRNA expression levels [37].

### Identification of the orthologous genes expressing orthologous circRNAs

**Fig. 3:**
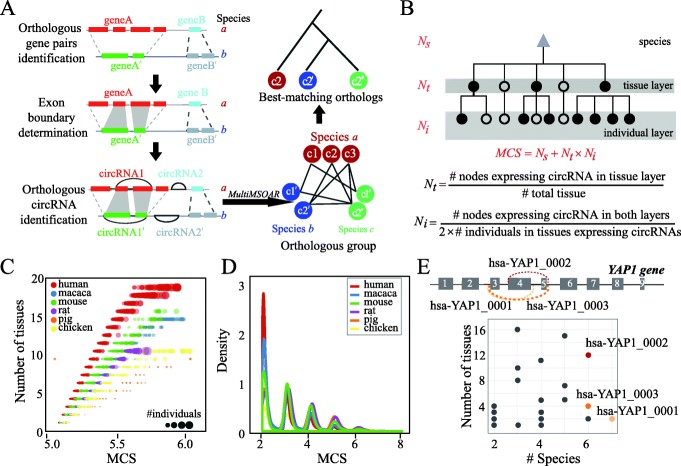
Novel quantitative assay for circRNA conservation analysis. **a** Workflow of the conserved circRNA identification. First, orthologous gene pairs were downloaded from the OMA database. Only orthologous gene pairs expressing circRNAs were retained. Next, the boundary of the cirexon pairs, defined as the exons involved in circRNA formation, in the orthologous gene pairs was determined based on whole genome alignments. To detect conserved circRNAs derived from these cirexons, 50-bp fragments on both sides of the circRNA BSJ were extracted and used to represent the BSJ sequence. All circRNA BSJ sequences from a single species were aligned to those from the other species using BLAT, followed by the reciprocal best hits strategy, to identify the orthologous circRNAs. Then, the MultiMSOAR tool was employed to integrate the reciprocal best pairwise orthologous circRNAs and obtain the best-matching orthologous circRNAs. **b** Scheme for the MCS calculation, integrating detailed conservation profiles in species, tissue, and individual cellular levels. The solid circles in each layer denote the tissues or individuals expressing the circRNA. **c** The distribution of MCS on highly conserved circRNAs with MCS scores ranging from 5 to 6. The scheme was able to successfully distinguish between the conservation of circRNAs either in species, tissues, or individual cells. Each circle denotes a circRNA and the circle size represents the number of individuals expressing this circular transcript. **d** Distribution of MCS for the conserved circRNAs identified in the six species. **e** Illustration of the improved candidate circRNA prioritization based on MCS using the *YAP1* gene. Top: illustration of the genomic region of the circRNAs derived from the human *YAP1* gene. Note that a total of 65 circRNAs were expressed from this gene but only the top 3 circRNAs (based on their MCS) were plotted. Curved gray lines indicate the BSJs of the three circRNAs. Bottom: conservation profiles of the 65 circRNAs derived from the *YAP1* gene. Each dot represents a circRNA. The gray, yellow, orange, and red colors represent increased MCS

Evolutionary analysis is crucial for insights into the genetic basis of functional screening. Therefore, we identified the evolutionarily conserved circRNAs, based on the orthologous genes, among these six species (Fig. 2b). This work was performed utilizing procedures that have been described previously [24]. In brief, a pairwise orthologous gene list was downloaded from the OMA ortholog database [38], and the circRNAs located on these orthologous genes for each species were determined using the genome annotation file. Subsequently, only the orthologous gene pairs in which both the members expressed the circRNAs were retained for the conserved circRNA detection. Next, the boundary of the cirexon pairs, defined as the exons involved in circRNA formation, in the orthologous gene pairs was determined from the whole genome alignments. These alignments, downloaded from UCSC and Ensembl, were used to ensure the accuracy of the conserved circRNA identification in downstream analysis. To detect the conserved circRNAs derived from these cirexons, 50-bp fragments on both sides of the circRNA BSJ were extracted from its corresponding cirexons and used to represent the BSJ sequence. All the circRNA BSJ sequences in one species were aligned to those in the other species using BLAT, followed by the reciprocal best hit strategy to find the orthologous circRNAs. Then, the MultiMSOAR v2.0 software [39], which uses a combinational approach to construct ortholog groups, was used to integrate the reciprocal best pairwise orthologous circRNAs. Finally, the resulting best-matched orthologous circRNAs were screened for downstream analysis (Fig. 3a). By performing this pipeline on the circRNAs from each of the six species, 129,635 circRNAs evolutionarily conserved between any two species were identified, 797 of which were conserved across all the six species.

### Analysis of the circRNA conservation levels

As conservation has often been considered a criterion to prioritize the functional candidate genes, we proposed the use of MCS to assess the conservation of the circRNA at the species, tissue, and individual (or sample) levels (Fig. 3b). This is calculated by the formula: MCS = *N*_*s*_ + *N*_*t*_ × *N*_*i*_. An example is shown in Additional file 1: Figure S1. Briefly, for a given circRNA in a species, this formula contains two parts of number: the integer part and the decimal part. The integer part, representing the conservation of circRNAs across species, is the number of orthologs of this circRNA (*N*_*s*_). The decimal part, *N*_*t*_ × *N*_*i*_, representing the conservation of circRNAs across tissues and individuals, is calculated by constructing a hierarchical expression tree, in which the circRNA expression pattern across tissues and individuals can be represented by the hierarchical structure within the two layers. The nodes in the tissue and individual layers represent the tissues and individuals in a particular species, respectively. Then, the score of this decimal part is calculated by *N*_*t*_ × *N*_*i*_, where

$$ {N}_t=\frac{\left(\# nodes\ expressing\ circRNA\ in\ the\ tissue\ layer\right)}{\left(\# total\ tissue\right)}, $$

$$ {N}_i=\frac{\left(\# nodes\ expressing\ circRNA\ in\ both\ layers\right)}{\left(2\times \# individuals\ in\ tissues\ expressing\ circRNA\right)}. $$

### Functional annotations of the circRNAs

As previously reported, functional annotation of the circRNAs using the comparative genomics approach has a limited usage, due to the rapid evolution of a vast majority of the circRNAs with unknown functions. To have a large-scale annotation of the circRNA function, we constructed a network for each species by integrating a number of interactions, including circRNA-miRNA, circRNA-mRNA, and circRNA-RBP. First, a Pearson correlation coefficient matrix for the circRNA-to-mRNA comparisons was constructed, based on the similarity between individual gene expression profiles. This was followed by applying a set of weak correlation filters, where *r* < 0.5 for gene pairs containing the circRNAs. To avoid false positives in the co-expression network analysis, the nodes on the network were restricted to mRNAs or the circRNAs expressed in at least 3 tissues. Next, miRanda v3.3a [40], TargetScan v7.0 [41, 42], and PITA v2.1.2 [43] were used to predict circRNA-miRNA interactions. The CLIP-Seq datasets from the starBase v2 [22] and the POSTAR [44] databases were utilized to predict the circRNA-RBP interactions, which were inferred by searching the RBP-binding peaks that were found within 1-kb regions on both sides of the BSJs. Finally, these three types of interactions were integrated and used for the GO and the KEGG annotations of the circRNAs.

## Utility and discussion

### A comprehensive compendium of the vertebrate circRNAs

To determine the circRNA repertoires of the six vertebrates, we analyzed 1070 samples (an average of 178 samples for each species) and approximately 40 billion reads, representing the transcriptomes of 19 different tissues or organs. Notably, the circRNAs from the macaque species were unique to the circAtlas, which will greatly expand the landscape of circRNAs and facilitate their conservation analysis. Owing to the contribution of the SEQC project [45], samples from the rat species largely outnumbered those from other species and contributed to more than 50% of the total samples. The combination of different algorithms has been shown to significantly improve the reliability of the identified circRNAs [31]. To this end, we used four different tools to detect the circRNAs from the RNA-seq datasets, including CIRI2, DCC, find_circ, and CIRCexplorer2. CircRNAs supported by at least two independent BSJ reads and detected by at least two tools were retained for downstream analysis. We identified 413,657, 169,618, 175,273, 80,158, 75,953, and 92,428 circRNAs for each species namely human, macaque, mouse, rat, pig, and chicken, respectively. Notably, the largest number of samples from rat did not result in the largest number of circRNAs, as most of these datasets were generated using 50-bp single-end RNA sequencing (Fig. 2a). Subsequently, the full-length circRNAs were assembled using the CIRI-full pipeline. This step produced 333,856, 144,992, 135,046, 71,052, 61,946, and 71,911 full-length circRNA for each species, respectively.

Compared to existing circRNA databases, circAtlas represents the most comprehensive repository of circRNAs in vertebrates. This is because circAtlas is characterized not only by the number of vertebrates but also by the number of detected circRNAs in normal tissues and multiple individuals. For example, circRNAs detected in mouse was an order of magnitude larger than any other databases, whereas this gap increased to two orders of magnitude in the total number of all the species, excluding human and mouse. The increased diversity of samples in circAtlas would allow simultaneous analysis across different dimensions, such as investigation of variations in circRNA expression patterns across individuals and tissues, even species. Moreover, each of the circRNAs annotated in circAtlas was identified by at least two detection tools and most of them were assembled into full-length transcripts, which further demonstrates the high quality of these circRNAs.

Normalized by the total mapped BSJ reads for each species, we revealed that the number of circRNAs significantly increased along with species evolution, in overall agreement with previous studies [46]. Next, we evaluated the selective constraints of circRNAs in terms of sequence conservation across species. The BLAT tool was used to determine the conservation of circRNAs among these six species. Using this method, we found that the vast majority of circRNAs (an average of 61.7%) could be detected only in one species, with only 797 circRNAs shared by all species, in agreement with previous reports on the highly species-specific expression of circRNAs (Fig. 2b). Further, the number of conserved circRNAs increased with reducing evolutionary distance among species.

### Improved resolution for the circRNA conservation analysis

For a comprehensive investigation of the circRNA conservation profiles, we used MCS to evaluate the conservation of the circRNAs across different species, tissues, or individuals. Briefly, for each circRNA, the MCS was obtained by adding the product of the normalized occurrences in tissue and individuals and the ortholog number of the corresponding circular transcript (Fig. 3b). To validate the performance of this method, we investigated the MCS distribution of the circRNAs in each species in a window between 5 and 6 (Fig. 3c). As a result, we observed a clear tendency of the MCS to be positively correlated to the circRNA conservation in species, tissue, and individual levels (Additional file 1: Figure S2). Same trends were also observed for other randomly selected windows. These results together showed that the MCS represented an effective parameter for analysis of the circRNA conservation.

Upon successfully assigning the conservation scores to the circRNAs, the distribution of the MCS scores in each species was profiled. We found that the circRNA transcription evolved rapidly and was expressed in a single species or closely related ones, where a large proportion of the circRNAs in each species had MCS lower than 2.0 (Fig. 3d). Collectively, these results showed that the MCS scheme enabled an increased resolution for the circRNA conservation analysis and will greatly facilitate the prioritization of the candidate circRNAs. As an example, 65 circRNAs derived from the *YAP* gene had been recorded in the circAtlas database (Fig. 3e). We ranked these circRNAs in terms of their MCS and found that the most conserved one, hsa-YAP1_0001, was expressed across 5 vertebrates and 12 tissues, suggesting that it may be associated with important biological functions. Indeed, previous studies have shown that the translation of YAP was antagonized by hsa-YAP1_0001 via suppression of the assembly of the translation initiation machinery [47].

### Comprehensive investigation of the circRNA subclasses

**Fig. 4:**
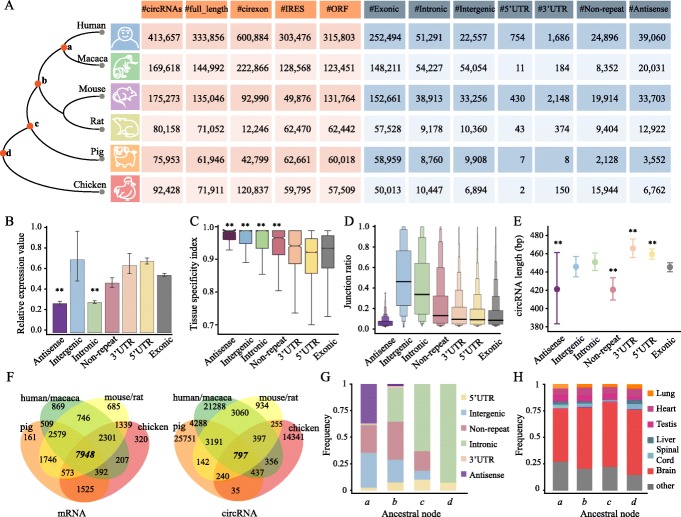
Characterization of different subclasses of circRNAs. **a** CircRNAs identified in the six vertebrates by CIRI2 and other tools. The first five columns show the numbers of identified circRNAs, full-length circRNAs, cirexons, IRES, and ORFs, respectively. The remaining columns indicate the number of circRNAs from the different subclasses. **b**–**e** The relative expression value (**b**), the tissue specificity index (**c**), the junction ratio across tissues (**d**), and the length distribution (**e**) of different types of circRNAs. Significance has been calculated between exonic and other types of circRNAs using the Wilcoxon test (**P < 0.01). **f** The Venn plot shows the number of shared genes and circRNAs across vertebrates. **g** The proportion of circRNA subclasses (except exonic ones) in different ancestral nodes (denoted by *a*, *b*, *c*, and *d*). **h** The proportion of circRNAs expressed in specific tissues across the different ancestral nodes

To better understand their diversity, the circRNAs were divided into seven groups according to their overlap with the known genetic components and the transcriptional direction, including exonic, intronic, intergenic, 5′-UTR, 3′-UTR, non-repeat, and the antisense regions. Notably, we introduced a new category of the circRNAs termed as non-repeat, where no known repeats (as recorded in the RepeatMasker database) were present on the two flanking introns of a certain circRNA. This type of circRNAs was possibly generated by the binding of RBPs, rather than by the matching of flanking repetitive sequences in introns [10, 24, 48]. We first evaluated the composition of the identified circRNAs based on this classification. Consistent with previous findings, the circRNAs predominantly originated from the annotated exons, with an evident preference towards coding sequences and 5′-UTR exons (Fig. 4a). We further performed detailed analyses of the expression patterns and the sequence length of these seven types of circRNAs. In this context, it is noteworthy that the exonic circRNAs were used as a control across the comparisons, unless otherwise specified. In principle, the antisense and the intron-containing circRNAs had the lowest expression levels among all the compared cases (Fig. 4b–e). Moreover, this measurement did not offer any quantitative distinction from the other types of circRNAs. However, these types of circRNAs were clearly distinguished in terms of the tissue specificity, the junction ratio, and the sequence length in human and other organisms. Among the compared subclasses, the antisense circRNAs were the most distinct and displayed the highest level of tissue specificity, the lowest level of junction ratio, and the greatest variation in sequence length. Considering that antisense circRNAs are transcribed in an orientation opposite to their host genes and may function as regulators of the sense mRNA, we hypothesized that their expression patterns may be highly correlated to their host genes. However, we found that most of the Pearson correlation coefficients between the antisense circRNAs and their host genes varied between − 0.1 and 0.5, an indicator of either no or low relatedness. The non-repeat circRNAs maintained moderate levels of expression and the tissue specificity across all compared cases, except those with the shortest sequence length.

We subsequently investigated the mechanism by which these different subclasses of the circRNAs were retained during evolution. We examined the orthologous circRNAs present in these six vertebrates and calculated the number of orthologous circRNAs that possessed the same sequences and the BSJs and the number of shared genes on their ancestral nodes. As shown in Fig. 4f, the number of shared orthologous circRNAs and the BSJs decreased rapidly with increased genetic distance among the species. In contrast, the number of shared orthologous mRNAs decreased much more slowly, indicating that although derived from the same genes, the circRNAs of different species diverged rapidly in terms of sequences and the BSJs. Then, the number of each orthologous circRNA subclass across these nodes was calculated, along with the proportion of each subclass during evolution. As shown in Fig. 4g, the proportion of antisense subclass dropped sharply, followed by an intergenic, non-repeat, and 3′-UTR regions. A number of intronic circRNAs were highly conserved across these organisms, even for distantly related species, exhibiting stronger selective constraints at the transcriptional level. Furthermore, we investigated whether the expression patterns of circRNAs in these nodes varied across tissues, and found that the circRNAs were highly tissue-specific and dominantly expressed in the brains (Fig. 4h).

### Large-scale functional annotation of the circRNAs

**Fig. 5:**
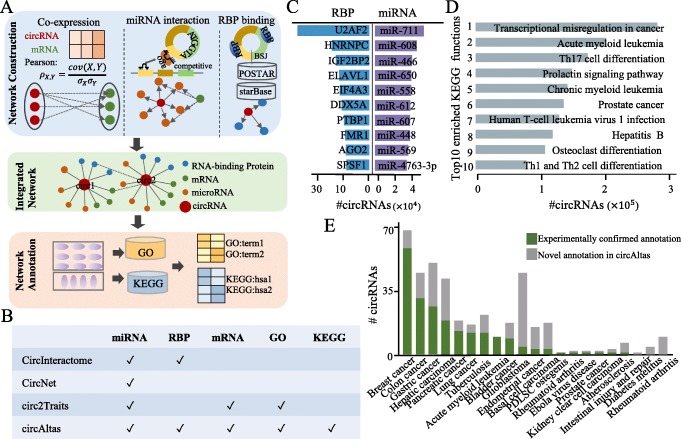
CircRNA annotation based on an integrated network of circRNAs, mRNAs, miRNAs, and RBPs. **a** Workflow of network construction. Briefly, the co-expression network was initially built based on the expression correlation between circRNAs and mRNAs. Then, circRNA-miRNA and circRNA-RBP interactions were added to the existing co-expression network. Finally, the potential GO and the KEGG functions of the circRNAs were predicted using the connected mRNAs, miRNAs, and RBPs. **b** Comparison of circAtlas with other circRNA annotation databases. **c** The top 10 most frequently interacting RBPs and miRNAs in the network. **d** The top 10 annotated KEGG pathways of the circRNAs. **e** Validation of accuracy of the circAtlas annotation using reported circRNAs. The *x*- and *y*-axes indicate the types of diseases and the number of circRNAs that have been reported (green) to be associated with diseases or have novel annotations (gray), respectively

The circRNAs have been demonstrated to act as sponges for miRNAs or RBPs and have been thus predicted to function as the post-transcriptional regulators of gene expression [49]. Moreover, the joint analysis of the large-scale transcriptomic data coupled with computational algorithm represents a powerful approach to elucidate possible biological roles of these genes. To identify functional circRNAs and predict their potential regulatory mechanisms, we integrated co-expression profiles and the circRNA-miRNA and the circRNA-RBP interaction networks to annotate the identified circRNAs in each species (Fig. 5a). Specifically, we inferred the co-expression networks independently for each of the six species, where nodes represented genes (circRNAs or mRNAs) and edges represented a co-expression relationship between the gene pairs. Then, the circRNA-miRNA interactions were added to the network by predicting the miRNA-binding site on the circRNAs. Moreover, the circRNA-RBP interactions were integrated into the network by searching for RBP-binding peaks around the BSJs based on the CLIP-Seq data. Finally, we inferred the potential functions for the circRNAs by utilizing the connected nodes and their annotated GO and KEGG terms. Compared with previous circRNA databases, the circAtlas integrated more functional genomic resources for the annotation of the circRNAs (Fig. 5b).

After constructing the network, we first surveyed those RBPs or miRNAs that may be able to interact with the circRNAs. Then, these RBPs and miRNAs were ranked by the number of the circRNAs associated with them. We found that the top 10 RBPs were highly enriched in the positive regulation function of transcription, such as U2AF2 and PTBP1 (Fig. 5c). The miRNAs that most frequently bound to the circRNAs were identified to be hsa-miR-711 and hsa-miR-608, which may assume pivotal roles in the circRNA regulation. Notably, most of the circRNAs were found to possess a very limited number of binding sites for different miRNAs, as opposed to the multiple binding sites for the same miRNA. We then extracted the top 10 most abundant KEGG terms and found that the largest number of annotated circRNAs was involved in disease-related functions, such as the transcriptional misregulation in cancer, leukemia, and prostate cancer (Fig. 5d). This suggests that circRNAs may play important regulatory roles in disease conditions. To further demonstrate the reliability of the circRNA annotations by the circAtlas, we compared them to the manual curations of the disease-associated circRNAs recorded in circad, circR2Disease, and circRNADisease. As a result, we found 456 circRNAs were shared among these databases, most of which were well-supported by previous experimental annotations (Fig. 5e).

## Conclusion

In this study, the circAtlas database was created by integrating a total of 1,007,087 circRNAs from six vertebrates (human, macaque, mouse, rat, pig, and chicken), of which more than 80% were successfully assembled into full-length circRNAs. Based on this large-scale circRNA dataset, we explored the expression pattern and evolutionary conservation of the circRNAs using a novel MCS scheme. We further constructed a regulatory network of the circRNAs by combining the circRNA-mRNA, circRNA-miRNA, and circRNA-RBP interactions. This network was subsequently utilized to predict the potential functions of the circRNAs based on the GO and the KEGG pathway analyses. The circAtlas is a versatile circRNA database, which provides detailed information on the circRNAs, including the full-length sequence, the expression pattern, the conservation, and the interaction with mRNAs, RBPs, and microRNAs. The prioritization of the essential circRNAs in the circAtlas based on these informative features will be of great help for the functional screening of circRNAs.

The decrease in financial burden associated with RNA sequencing has led to a staggering increase in the number of identified circRNAs. However, present circRNA databases face challenges in terms of either the low number of species and tissues included as the circRNA sources or the absence of functional annotations. These bottlenecks greatly limit the application of such methods to the candidate circRNA prioritization for multiple reasons. First, the collection of samples from different tissues, cell lines, or tissues in different physiological conditions may lead to a biased analysis of the circRNA expression profiles. Second, the scarcity of the circRNA profiles across multiple species translates to the inability to screen the evolutionarily conserved circRNAs. In addition, the functional annotation of circRNAs largely relies on their host gene annotation. However, the circAtlas outperforms previous databases due to the following aspects: (i) it provides the most comprehensive circRNA expression landscapes thus far, based on 19 healthy tissues collected from 6 vertebrates; (ii) it presents a novel, comprehensive view of 818,803 full-length circRNAs in vertebrates and improves our understanding on the composition and the evolutionary conservation of these circRNAs; and (iii) it increases the resolution for the circRNA conservation analysis by employing the MCS-based scheme. Detailed conservation profiles of the candidate circRNAs can be obtained using circAtlas, not only across species but also across different tissues and individuals. In addition, in-depth functional annotations based on the integrated circRNA regulatory networks, including the co-expression and the microRNA- and the RBP-binding profiles, could be performed. Taken together, the present report of the development of the circAtlas represents a substantial advancement in the annotation and functional prioritization of circRNAs.

## Supplementary information

**Additional file 1: Figure S1.** An example shows the calculation of the MCS score. **Figure S2.** The correlation between the MCS scores of circRNAs and the conservation scores of their corresponding linear counterparts. **Table S1.** RNA-seq datasets used in this study. **Table S2.** Comparison between circAtlas1.0 and circAtlas 2.0. **Table S3.** Bioinformatic softwares and parameter settings used in this study.

**Additional file 2.** Review History.

## Data Availability

The circRNAs identified in this study with their annotations and full-length sequences can be directly downloaded at http://circatlas.biols.ac.cn/. The RNA-seq datasets used for circRNA identification are listed in Additional file 1: Table S1.
