# Supplementary material for: CircAtlas: an integrated resource of one million highly accurate circular RNAs from 1070 vertebrate transcriptomes
Source: Genome Biol. 2020 Apr 28;21:101. doi: 10.1186/s13059-020-02018-y (PMC7187532; doi:10.1186/s13059-020-02018-y)
Supplement: Supplementary file 1 — Additional file 1: Figure S1. An example shows the calculation of the MCS score. Figure S2. The correlation between the MCS scores of circRNAs and the conservation scores of their corresponding linear counterparts. Table S1. RNA-seq datasets used in this study. Table S2. Comparison between circAtlas1.0 and circAtlas 2.0. Table S3. Bioinformatic softwares and parameter settings used in this study. [file 13059_2020_2018_MOESM1_ESM.docx]

**Figure S1. An example shows the calculation of the MCS score.**

**
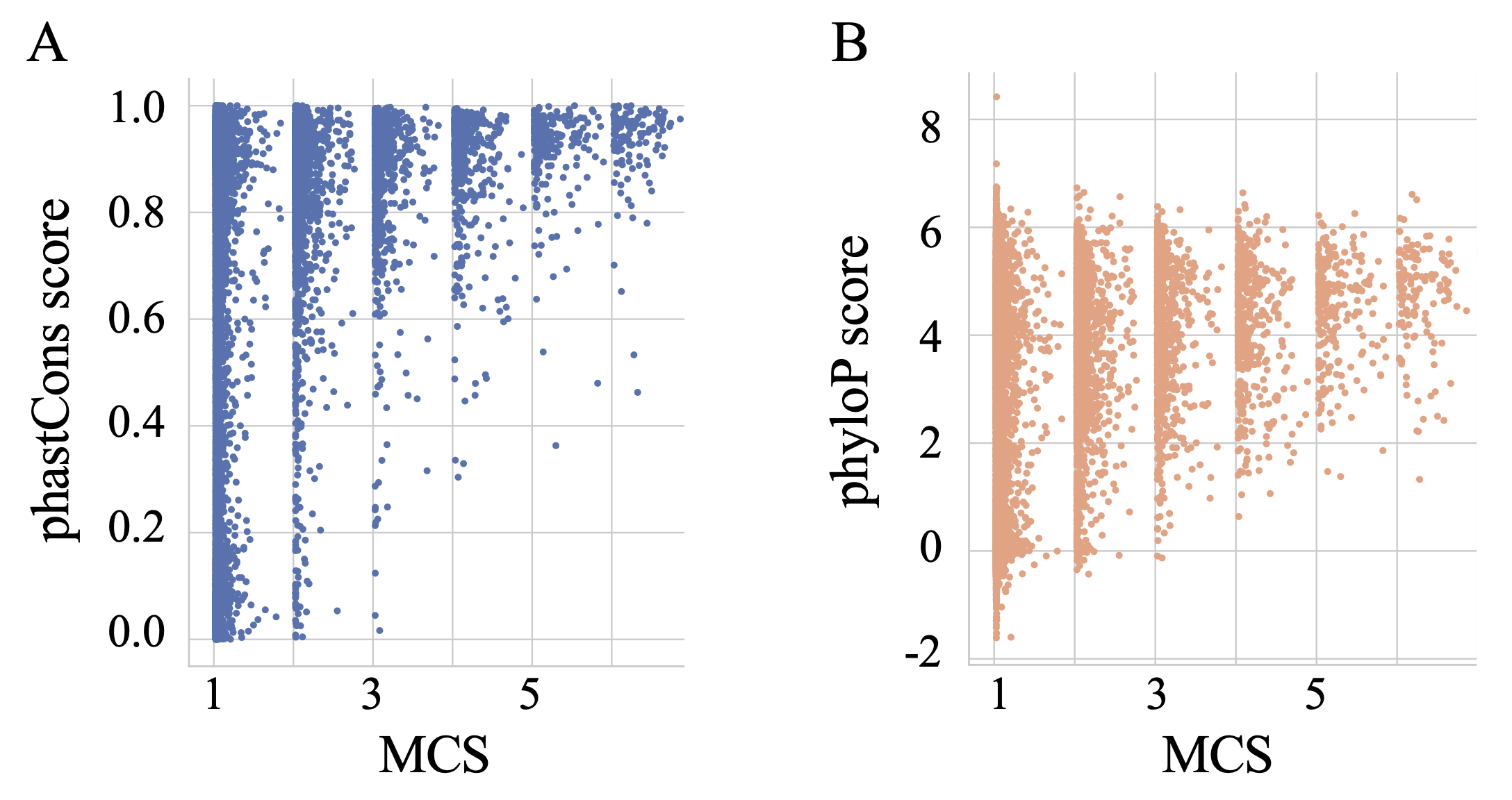
**

**Figure S2. The correlation between the MCS scores of circRNAs and the conservation scores of their corresponding linear counterparts.** (A) phastCons. (B) phyloP. Each dot represents a circRNA.

**Table S1. RNA-seq datasets used in this study.**

| **Species** | **Tissue** | **SRA number** |
| --- | --- | --- |
| pig | Heart | SRR2564757 |
| pig | Testis | SRR2564762 |
| pig | Spleen | SRR2564759 |
| pig | Skeletal Muscle | SRR2564767,SRR2564772,SRR2564771,SRR2564768,SRR2564774,  SRR2564770,SRR2564769 |
| pig | Brain | SRR2148913 |
| pig | Lung | SRR2564760 |
| pig | Liver | SRR2564758 |
| pig | Kidney | SRR2564761 |
| rat | Heart | SRR1170066,SRR1170061,SRR1170052,SRR1170051,SRR1170025,  SRR1170038,SRR1170055,SRR1170065,SRR1170074,SRR1170029,  SRR1170033,SRR1170034,SRR1170044,SRR1170047,SRR1170070,  SRR1170077,SRR1170078,SRR1170080,SRR1170087,SRR1170088,  SRR1170084,SRR1170073,SRR1170062,SRR1170043,SRR1170040,  SRR1170037,SRR1170026,SRR1170083,SRR1170069,SRR1170059,  SRR1170056,SRR1170048,SRR1170030,SRR1170085,SRR1170082,  SRR1170075,SRR1170072,SRR1170068,SRR1170058,SRR1170042,  SRR1170041,SRR1170039,SRR1170036,SRR1170031,SRR1170032,  SRR1170045,SRR1170046,SRR1170067,SRR1170076,SRR1170081,  SRR1170090,SRR1170027,SRR1170049,SRR1170054,SRR1170057,  SRR1170063,SRR1170064,SRR1170089,SRR1170079,SRR1170071,  SRR1170060,SRR1170053,SRR1170050,SRR1170035,SRR1170028 |
| rat | Testis | SRR1170492,SRR1170488,SRR1170487,SRR1170517,SRR1170510,  SRR1170495,SRR1170518,SRR1170509,SRR1170506,SRR1170501,  SRR1170499,SRR1170491,SRR1170513,SRR1170502,SRR1170496,  SRR1170514,SRR1170505,SRR1170494,SRR1170504,SRR1170503,  SRR1170516,SRR1170507,SRR1170493,SRR1170515,SRR1170512,  SRR1170497,SRR1170511,SRR1170508,SRR1170500,SRR1170498,  SRR1170490,SRR1170489 |
| rat | Spleen | SRR1170395,SRR1170362,SRR1170361,SRR1170356,SRR1170411,  SRR1170396,SRR1170416,SRR1170403,SRR1170392,SRR1170378,  SRR1170412,SRR1170369,SRR1170366,SRR1170359,SRR1170400,  SRR1170383,SRR1170380,SRR1170377,SRR1170374,SRR1170388,  SRR1170419,SRR1170415,SRR1170399,SRR1170370,SRR1170373,  SRR1170384,SRR1170387,SRR1170391,SRR1170355,SRR1170365,  SRR1170358,SRR1170368,SRR1170371,SRR1170402,SRR1170398,  SRR1170386,SRR1170385,SRR1170372,SRR1170418,SRR6240076,  SRR1170401,SRR1170420,SRR1170410,SRR1170382,SRR1170381,  SRR1170376,SRR1170375,SRR1170357,SRR6240079,SRR1170414,  SRR1170413,SRR1170393,SRR1170390,SRR1170389,SRR1170379,  SRR1170367,SRR1170364,SRR1170417,SRR1170409,SRR1170360,  SRR1170363,SRR1170394,SRR1170397 |
| rat | Thymus | SRR1170421,SRR1170434,SRR1170455,SRR1170452,SRR1170478,  SRR1170470,SRR1170465,SRR1170433,SRR1170426,SRR1170448,  SRR1170480,SRR1170484,SRR1170473,SRR1170469,SRR1170466,  SRR1170459,SRR1170444,SRR1170443,SRR1170437,SRR1170422,  SRR1170483,SRR1170474,SRR1170461,SRR1170451,SRR1170440,  SRR1170438,SRR1170430,SRR1170429,SRR1170425,SRR1170477,  SRR1170462,SRR1170456,SRR1170447,SRR1170427,SRR1170446,  SRR1170441,SRR1170432,SRR1170428,SRR1170463,SRR1170481,  SRR1170476,SRR1170435,SRR1170445,SRR1170454,SRR1170471,  SRR1170468,SRR1170464,SRR1170458,SRR1170479,SRR1170486,  SRR1170482,SRR1170475,SRR1170460,SRR1170457,SRR1170450,  SRR1170449,SRR1170439,SRR1170431,SRR1170424,SRR1170485,  SRR1170472,SRR1170467,SRR1170453,SRR1170442,SRR1170436,  SRR1170423 |
| rat | Skeletal Muscle | SRR1170297,SRR1170312,SRR1170326,SRR1170325,SRR1170315,  SRR1170338,SRR1170351,SRR1170321,SRR1170311,SRR1170300,  SRR1170298,SRR1170289,SRR1170348,SRR1170347,SRR1170340,  SRR1170333,SRR1170330,SRR1170329,SRR1170319,SRR1170304,  SRR1170303,SRR1170294,SRR1170293,SRR1170307,SRR1170316,  SRR1170334,SRR1170337,SRR1170343,SRR1170352,SRR1170290,  SRR1170308,SRR1170322,SRR1170344,SRR1170291,SRR1170306,  SRR1170301,SRR1170299,SRR1170336,SRR1170335,SRR1170309,  SRR1170342,SRR1170345,SRR1170346,SRR1170332,SRR1170331,  SRR1170313,SRR1170302,SRR1170296,SRR1170354,SRR1170353,  SRR1170323,SRR1170320,SRR1170317,SRR1170310,SRR1170292,  SRR1170295,SRR1170305,SRR1170314,SRR1170318,SRR1170324,  SRR1170327,SRR1170328,SRR1170339,SRR1170341,SRR1170349,  SRR1170350 |
| rat | Brain | SRR1170022,SRR1170016,SRR1170015,SRR1170008,SRR1169998,  SRR1169960,SRR1169963,SRR1169994,SRR1169997,SRR1170000,  SRR1170003,SRR1170019,SRR1170007,SRR1170004,SRR1169964,  SRR1170021,SRR1170011,SRR1170006,SRR1170005,SRR1169961,  SRR1169962,SRR1169995,SRR1169996,SRR1170001,SRR1170002,  SRR1170023,SRR1169959,SRR1169999,SRR1170010,SRR1170013,  SRR1170020,SRR1170024,SRR1170018,SRR1170017,SRR1170014,  SRR1170009, SRR1170012 |
| rat | Lung | SRR1170190,SRR1170179,SRR1170197,SRR1170167,SRR1170160,  SRR1170214,SRR1170213,SRR1170185,SRR1170194,SRR1170186,  SRR1170218,SRR1170202,SRR1170205,SRR1170198,SRR1170171,  SRR1170176,SRR1170181,SRR1170182,SRR1170175,SRR1170168,  SRR1170164,SRR1170158,SRR1170193,SRR1170201,SRR1170210,  SRR1170220,SRR1170172,SRR1170163,SRR1170157,SRR1170189,  SRR1170206,SRR1170209,SRR1170217,SRR1170184,SRR1170183,  SRR1170200,SRR1170174,SRR1170173,SRR1170169,SRR1170159,  SRR1170207,SRR1170222,SRR1170219,SRR1170215,SRR1170204,  SRR1170199,SRR1170196,SRR1170161,SRR1170170,SRR1170187,  SRR1170221,SRR1170216,SRR1170211,SRR1170208,SRR1170162,  SRR1170165,SRR1170195,SRR1170188,SRR1170192,SRR1170191,  SRR1170180,SRR1170178,SRR1170177,SRR1170166,SRR1170203,  SRR1170212 |
| rat | Liver | SRR1170275,SRR1170282,SRR1170224,SRR1170231,SRR1170239,  SRR1170257,SRR1170250,SRR1170249,SRR1170260,SRR1170272,  SRR1170267,SRR1170253,SRR1170242,SRR1170281,SRR1170276,  SRR1170263,SRR1170241,SRR1170246,SRR1170232,SRR1170228,  SRR1170227,SRR1170235,SRR1170245,SRR1170254,SRR1170258,  SRR1170264,SRR1170268,SRR1170271,SRR1170279,SRR1170286,  SRR1170223,SRR1170285,SRR1170243,SRR1170237,SRR1170244,  SRR1170259,SRR1170266,SRR1170269,SRR1170273,SRR1170284,  SRR1170283,SRR1170274,SRR1170261,SRR1170251,SRR1170240,  SRR1170238,SRR1170230,SRR1170229,SRR1170225,SRR1170288,  SRR1170278,SRR1170287,SRR1170270,SRR1170265,SRR1170255,  SRR1170252,SRR1170234,SRR1170226,SRR1170233,SRR1170247,  SRR1170248,SRR1170256,SRR1170262,SRR1170277,SRR1170280 |
| rat | Uterus | SRR1170523,SRR1170520,SRR1170541,SRR1170539,SRR1170528,  SRR1170550,SRR1170545,SRR1170542,SRR1170536,SRR1170535,  SRR1170546,SRR1170532,SRR1170531,SRR1170549,SRR1170527,  SRR1170524,SRR1170530,SRR1170529,SRR1170519,SRR1170533,  SRR1170547,SRR1170540,SRR1170548,SRR1170534,SRR1170543,  SRR1170537,SRR1170552,SRR1170551,SRR1170538,SRR1170526,  SRR1170525,SRR1170544,SRR1170522,SRR1170521 |
| rat | Kidney | SRR1170150,SRR1170145,SRR1170124,SRR1170123,SRR1170114,  SRR1170109,SRR1170101,SRR1170099,SRR1170096,SRR1170091,  SRR1170106,SRR1170113,SRR1170127,SRR1170136,SRR1170139,  SRR1170102,SRR1170117,SRR1170118,SRR1170128,SRR1170132,  SRR1170135,SRR1170146,SRR1170153,SRR1170154,SRR1170149,  SRR1170141,SRR1170131,SRR1170120,SRR1170110,SRR1170105,  SRR1170095,SRR1170142,SRR1170092,SRR1170156,SRR1170143,  SRR1170137,SRR1170130,SRR1170112,SRR1170107,SRR1170098,  SRR1170100,SRR1170108,SRR1170115,SRR1170119,SRR1170125,  SRR1170129,SRR1170134,SRR1170144,SRR1170151,SRR1170093,  SRR1170094,SRR1170104,SRR1170111,SRR1170121,SRR1170126,  SRR1170138,SRR1170140,SRR1170148,SRR1170155,SRR1170152,  SRR1170147,SRR1170133,SRR1170122,SRR1170116,SRR1170103,  SRR1170097 |
| human | Placenta | BIGD ID: PRJCA000751,SRR6463513,SRR6463506,SRR6324444,  SRR6324443,SRR6463491,SRR6463499,SRR6463501,SRR6463509,  SRR6463514,SRR6463518,SRR6463524,SRR6463488,SRR6463492,  SRR6463495,SRR6463505,SRR6463510,SRR6463520,SRR6463523,  SRR6463517,SRR6463502,SRR6463496,SRR6463487,SRR6463525,  SRR6463522,SRR6463515,SRR6463508,SRR6463500,SRR6463498,  SRR6463497,SRR6463490,SRR6324445,SRR6463489,SRR6463493,  SRR6463494,SRR6463504,SRR6463507,SRR6463511,SRR6463512,  SRR6463521,SRR6463503,SRR6463516,SRR6463519 |
| human | Heart | SRR1721288,SRR4421689,SRR1721287,SRR1721284,SRR787274,  SRR1721286,SRR1721285,BIGD ID: PRJCA000751,SRR1721289,  SRR4421789,SRR3192433,SRR3192434,SRR1721290 |
| human | Testis | SRR4421668,SRR4421667,BIGD ID: PRJCA000751,SRR4422587,  SRR4422588 |
| human | Stomach | SRR4422210,SRR1721310,SRR4421597,SRR1721309,SRR3192450,  BIGD ID: PRJCA000751,SRR3192449,SRR4422373,SRR1721313,  SRR1721307,SRR1721311,SRR1721308,SRR1721312 |
| human | Spleen | BIGD ID: PRJCA000751,SRR4421642,SRR4421334,SRR4422445,  SRR4421468 |
| human | Bone | SRR5048184,SRR5048183,BIGD ID: PRJCA000751 |
| human | Thymus | BIGD ID: PRJCA000751 |
| human | Brain | SRR3192424,SRR1993678,SRR3151754,BIGD ID: PRJCA000751,  SRR1993677,SRR1993680,SRR3151753,SRR3192427,SRR3192463,  SRR3151757,SRR787271,SRR3151760,SRR3192432,SRR3192464,  SRR3192446,SRR3192445,SRR3192431,SRR3192428,SRR3151758,  SRR3151750,SRR3192444,SRR3151752,SRR1993676, SRR1993682,  zheng et al, Genome Medicine, 2019, 11:2,SRR1993679,SRR3151759  SRR3151755,SRR3151756,SRR3151761,SRR3192425,SRR3192443,  SRR3151751, |
| human | Small  Intestine | SRR1721296,SRR1721295,SRR1721291,SRR1721292,SRR1721293  BIGD ID: PRJCA000751,BIGD ID: PRJCA000751,SRR1721294,  SRR1721297, |
| human | Colon | SRR4421756,BIGD ID: PRJCA000751,SRR317086,SRR4422344,  SRR317093,SRR317094,SRR317090,SRR317089,SRR317091,  SRR787273,SRR317096,SRR4422147,SRR4422379,SRR317092,  SRR317095,SRR4421313,SRR4422057,SRR317087,SRR317088 |
| human | Liver | N11_GSM2053440,SRR3192439,N7_SRX1558028,SRR4421874,  N10_GSM2053439,SRR4421506,SRR787276,N20_GSM2053449,  N17_GSM2053446,N13_GSM2053442,N15_GSM2053444,  N19_GSM2053448,N22_GSM2053451,N8_GSM2053438,  N12_GSM2053441,N6_SRX1558027,N3_SRX1558026,  N26_GSM2053454,N24_GSM2053452,N21_GSM2053450,  N18_GSM2053447,N14_GSM2053443,BIGD ID: PRJCA000751,  SRR3192419,SRR3192440,N16_GSM2053445,N25_GSM2053453 |
| human | Spinal Cord | BIGD ID: PRJCA000751,SRR3192451,SRR3192452 |
| human | Uterus | SRR3192461,SRR4422656,BIGD ID: PRJCA000751,SRR3192462,  SRR4421350 |
| human | Lung | SRR1297309,SRR1721305,SRR1297305,SRR1721301,SRR1721306,  SRR3192441,SRR3192442,SRR4421631,SRR1721302,SRR1297306,  BIGD ID: PRJCA000751,SRR1721304,SRR4422346,SRR4421758,  SRR1721303,SRR1297308,SRR1297307,SRR1297304,SRR787277,  SRR4421779 |
| human | Pancreas | SRR4422136,SRR4422592 |
| human | Prostate | SRR4422158,BIGD ID: PRJCA000751,SRR4421868,SRR787280 |
| human | Skeletal Muscle | BIGD ID: PRJCA000751,SRR3192454,SRR3192453,SRR787278 |
| human | Kidney | SRR787275,SRR1721299,BIGD ID: PRJCA000751,SRR3192437,  SRR1721298,SRR1721300,SRR3192438 |
| macaca | Heart | BIGD ID: PRJCA000751 |
| macaca | Testis | BIGD ID: PRJCA000751 |
| macaca | Stomach | BIGD ID: PRJCA000751 |
| macaca | Spleen | BIGD ID: PRJCA000751 |
| macaca | Retina | Sun st al, RNA Biology 2019,4:2 |
| macaca | Skeletal Muscle | SRR2337335,SRR2337332,SRR2337331,SRR2337328,SRR2337327,  SRR2337324,SRR2337323,SRR2337320,SRR2337318,SRR2337317,  SRR2337314,SRR2337313,BIGD ID: PRJCA000751,SRR2337334,  SRR2337333,SRR2337330,SRR2337329,SRR2337326,SRR2337325,  SRR2337322,SRR2337321,SRR2337319,SRR2337315,SRR2337312 |
| macaca | Brain | BIGD ID: PRJCA000751,SRR5088736,SRR5088735,SRR5088728,  SRR5088727,SRR5088724,SRR5088723,SRR5088720,SRR5088719,SRR5088734,  SRR5088733,SRR5088730,SRR5088726,SRR5088725,SRR5088722,SRR5088721, |
| macaca | Lung | BIGD ID: PRJCA000751 |
| macaca | Liver | BIGD ID: PRJCA000751 |
| macaca | Spinal | BIGD ID: PRJCA000751 |
| macaca | Uterus | BIGD ID: PRJCA000751 |
| macaca | Prostate | BIGD ID: PRJCA000751 |
| macaca | Kidney | BIGD ID: PRJCA000751 |
| macaca | Pancreas | BIGD ID: PRJCA000751 |
| chicken | Kidney | ERR1298585,ERR1298582,ERR1298581,ERR1298579,ERR1298584,  ERR1298583,ERR1298580,ERR1298578 |
| chicken | Stomach | ERR1298632,ERR1298631,ERR1298628,ERR1298627,ERR1298633,  ERR1298630,ERR1298629,ERR1298626 |
| chicken | Spleen | ERR1298646,ERR1298645,ERR1298642,ERR1298648,ERR1298647,  ERR1298644,ERR1298643 |
| chicken | Thymus | ERR1298657,ERR1298654,ERR1298653,ERR1298650,ERR1298649,  ERR1298656,ERR1298655,ERR1298652,ERR1298651 |
| chicken | Brain | ERR1298536,ERR1298535,ERR1298532,ERR1298531,ERR1298590,  ERR1298589,ERR1298586,zheng et al, Genome Medicine, 2019,11:2, ERR1298537,ERR1298534,ERR1298533,ERR1298530,ERR1298592,  ERR1298591,ERR1298588,ERR1298587 |
| chicken | Lung | ERR1298609,ERR1298606,ERR1298605,ERR1298602,ERR1298608,  ERR1298607,ERR1298604,ERR1298603 |
| chicken | Liver | ERR1298598,ERR1298597,ERR1298594,ERR1298601,ERR1298596,  ERR1298595,ERR1298600,ERR1298599 |
| chicken | Heart | ERR1298568,ERR1298567,ERR1298564,ERR1298563,ERR1298569,  ERR1298566,ERR1298565,ERR1298562 |
| chicken | Skeletal Muscle | SRR4734704,SRR4734703,SRR4734706,SRR4734705,SRR4734702 |
| chicken | Pancreas | ERR1298623,ERR1298624,ERR1298620,ERR1298618,ERR1298625,  ERR1298621,ERR1298619 |
| mouse | Heart | SRR964800,SRR964799,SRR964796,SRR964795,SRR964792,  SRR964791,SRR3192548,SRR2927736,SRR1796709,SRR1796710,  SRR1772419,BIGD ID: PRJCA000751,SRR964802,SRR964801,  SRR964798,SRR964797,SRR964794,SRR964793,SRR964790,  SRR3192549,SRR1772417 |
| mouse | Testis | BIGD ID: PRJCA000751,SRR2927742,SRR1796716,SRR1796715,  SRR1772420 |
| mouse | Stomach | BIGD ID: PRJCA000751 |
| mouse | Spleen | SRR2927741,BIGD ID: PRJCA000751 |
| mouse | Thymus | BIGD ID: PRJCA000751,SRR2927743 |
| mouse | Skeletal Muscle | SRR3192323,SRR3192320,BIGD ID: PRJCA000751,SRR3192322,  SRR3192321,BIGD ID: PRJCA000751 |
| mouse | Brain | BIGD ID: PRJCA000751, SRR1772427,SRR1772424,SRR1772423,  SRR3192648,SRR3192588,SRR2927735,SRR1993674,SRR1993673,  SRR1993665,SRR1772430,SRR1772429,SRR1772426,SRR1772425,  SRR1772416,SRR3192589,SRR1993675,SRR1993672,SRR1993664,  SRR1796708,SRR1796707,SRR1772432,SRR1772431,SRR1772428, |
| mouse | Small Intestine | BIGD ID: PRJCA000751,SRR3192318,SRR3192317,  BIGD ID: PRJCA000751,SRR3192316 |
| mouse | Lung | BIGD ID: PRJCA000751,SRR2927739,SRR1796714,SRR1796713,  SRR1772418 |
| mouse | Liver | BIGD ID: PRJCA000751,SRR3192470,SRR3192469,SRR2927738,  SRR1796712,SRR1796711 |
| mouse | Spinal Cord | SRR1993670,SRR1993669,BIGD ID: PRJCA000751,SRR1993671,  SRR1993668,SRR1993667 |
| mouse | Colon | BIGD ID: PRJCA000751 |
| mouse | Prostate | BIGD ID: PRJCA000751 |
| mouse | Kidney | BIGD ID: PRJCA000751,SRR2927737 |
|  |  |  |
|  |  |  |

**Table S2. Comparison between circAtlas1.0 and circAtlas 2.0**.

|  | Cell Reports, 2019 | This Study |
| --- | --- | --- |
| **Statistics** |  |  |
| # organisms | 3 (Human, Macaque, Mouse) | 6 (Human, Macaque, Mouse, Rat, Pig, Chicken) |
| # tissue type | 17 | 19 |
| # RNA-seq data sets | 88 | 1070 |
| # identified circRNAs | 283,384 | 1,007,087 |
| # full-length circRNAs | 205,693 | 818,803 |
| Confidence of identified circRNAs | only CIRI2 was used | circRNAs were supported by at least two of the following tools (CIRI2, CIRCexplorer2, find_circ, DCC) |
| **Function annotation** | | |
| Multiple conservation score | No | Yes |
| KEGG annotation | No | Yes |
| GO annotation | No | Yes |
| Coding potential | No | Yes |
| Liftover across circRNAs databases | No | Yes |
| mRNA-circRNA network | constructed but not integrated | Yes |
| RBP binding network | No | Yes |
| miRNA binding network | No | Yes |
| **Homepage** |  |  |
| Web design | limited functions | Well designed with integrated resources |
| Data download | No | Yes |
| web link | http://zhaolab.biols.ac.cn | http://circatlas.biols.ac.cn |

**Table S3. Bioinformatic softwares and parameter settings used in this study**.

| **Software** | **Command line** |
| --- | --- |
| ComBat | combat_edata2=ComBat(dat=edata,batch=batch,mod=NULL,par.prior=FALSE, mean.only=TRUE) |
| CIRI2/CIRI-as/CIRI-full | java -jar -Xmx2028m CIRI-full-pipe v1.1.jar -1 R1.fq.gz -2 R2.fq.gz -r reference.fa -a genome.gtf -d out -t 6 -0 |
| CIRCexplor2 | bwa mem -T 19 -t 16 bwa_index R1.fq.gz R2.fq.gz > out.sam  CIRCexplorer2 parse -t BWA out.sam -b out_back_spliced_junction.bed > out_CIRCexplorer2_parse.log  CIRCexplorer2 annotate -r reference.fa -g genome.gtf -b out_back_spliced_junction.bed -o out_circularRNA_known.txt |
| find_circ | bowtie2 -p 16 --very-sensitive --score-min=C,-15,0 --mm -x bowtie_index -q -1 R1.fq.gz -2 R2.fq.gz -S out.sam  samtools view -bS out.sam \| samtools sort -o out.bam  samtools view -hf 4 out.bam \| samtools view -Sb -> unmapped.bam  python find_circ/unmapped2anchors.py unmapped.bam \| gzip >anchors.fastq.gz  rm -f out.sam\nrm -f out.bam\nrm -f unmapped.bam\nbowtie2 -p 16 --score-min=C,-15,0 --reorder --mm -q -U anchors.fastq.gz -x bowtie_index\| python find_circ/find_circ.py -G reference.fa -p out -s out/stats.txt R out spliced_reads.fa > out/find_circ.sites.bed |
| DCC | STAR --runThreadN 16 --genomeDir star_index --outSAMtype BAM SortedByCoordinate --readFilesIn R1.fq.gz R2.fq.gz --outFileNamePrefix out --outReadsUnmapped Fastx --outSJfilterOverhangMin 15 15 15 15 --alignSJoverhangMin 15 --alignSJDBoverhangMin 15 --outFilterMultimapNmax 20 --outFilterScoreMin 1 --outFilterMatchNmin --outFilterMismatchNmax 2 --chimSegmentMin 15 --chimScoreMin 15 --chimScoreSeparation 10 --chimJunctionOverhangMin 15  STAR --runThreadN 16 --genomeDir out --outSAMtype None -readFilesIn R1.fq.gz--outFileNamePrefix out_M1 --outReadsUnmapped Fastx  --outSJfilterOverhangMin 15 15 15 15 --alignSJoverhangMin 15 --alignSJDBoverhangMin 15 --seedSearchStartLmax 30 --outFilterMultimapNmax 20 --outFilterScoreMin 1 --outFilterMatchNmin 1 --outFilterMismatchNmax 2 --chimSegmentMin 15 --chimScoreMin 15 --chimScoreSeparation 10 --chimJunctionOverhangMin 15  STAR --runThreadN 16 --genomeDir {0} --outSAMtype None--readFilesIn R2.fq.gz --outFileNamePrefix out_M2 --outReadsUnmapped Fastx --outSJfilterOverhangMin 15 15 15 15 --alignSJoverhangMin 15 --alignSJDBoverhangMin 15 --seedSearchStartLmax 30 --outFilterMultimapNmax 20 --outFilterScoreMin 1 --outFilterMatchNmin 1--outFilterMismatchNmax 2 --chimSegmentMin 15 --chimScoreMin 15 --chimScoreSeparation 10--chimJunctionOverhangMin 15 |
| Gene expression | Hisat2 -p cpu -q -x reference.index -1 f1 -2 f2 \| samtools view -bS - > out.bam  samtools sort bamFile -o out_sorted.bam  stringTie out_sorted.bam -p 1 -G genome.gtf -o out/genes.gtf -l out -A gene_abund.out |
| IRESfinder | IRESfinder.py -f a -o out.result -m 2 -w 174 -s 50 |
| ORFfinder | python3 orf_finder.py -i full-length.fa -o out -min 30 -st both -num 100 |
| Ortholog circRNAs | Liftover chain.file bed1 bed.mapped bed.unmapped --minMatch=0.6  Blat fasta fast2 -out=blast9 out  MultiMSOAR2.0 #species speciesTree geneFamilyn-o GeneInfo -o OrthoGroup |
| Miranda | miranda mirna.fa full-length.fa -sc 140 -en -17 -out out |
| Pita | pita_prediction.pl -utr full-length.fa -mir mirna.fa -prefix out |
| TargetScan | targetscan_70.pl miR_Family_info_sample.txt UTR_Sequences_sample.txt targetscan_70_output.txt |
| Annotation | library(clusterProfiler)  GO<-enrichGO(genelist,org.Hs.eg.db, ont='ALL',pAdjustMethod = 'BH',pvalueCutoff = 0.05,qvalueCutoff = 0.1,keyType = 'ENSEMBL')  kegg<- enrichKEGG(gene,organism=’hsa’,keyType = "kegg", pvalueCutoff = 0.05,qvalueCutoff = 0.1,pAdjustMethod = 'BH',use_internal_data =T) |
