# Supplementary material for: CircAtlas: an integrated resource of one million highly accurate circular RNAs from 1070 vertebrate transcriptomes
Source: Genome Biol. 2020 Apr 28;21:101. doi: 10.1186/s13059-020-02018-y (PMC7187532; doi:10.1186/s13059-020-02018-y)
Supplement: Supplementary file 2 — Additional file 2. Review History. [file 13059_2020_2018_MOESM2_ESM.docx]

**Review History**

**First round of review**

**Reviewer 1**

Wu, Ji, and Zhao developed a comprehensive database, CircAltas2.0, based on 1,070 RNA-seq samples from 19 normal tissues across six species. The authors claimed that this database contains 1,014,931 highly confident circRNAs and >80% are as-sembled into full-length sequences. This database is an extended version of its pre-vious one (CircAltas1.0). The new version provides in-depth information such as conservation and functional annotation. The database is well organized and us-er-friendly. However, there are some concerns listed below.

Major Points:
1.     Four circRNA detection tools (CIRI2, find_circ, CIRCexploere2, and DCC) were used in this database. There are dozens of publicly available circRNA detectors. The reasons why the authors selected these four tools should be provided.
2.     The authors claimed that the identified circRNAs were "highly confident". The authors should provide lines of evidence to support their claim (i.e., "highly con-fident" circRNAs). For example, do they use some strategies to further remove false positives that were derived from ambiguous alignments?
3.     The definition of multiple conservation score (MCS) is arbitrary. How did the authors define the weighted coefficients for Ns, Nt, and Ni? Undoubtedly, ac-cording to their definition, the cis-backsplicing junctions detected from human would have the larger MCS scores as compared with those detected from other species because of much more available samples (tissues/individuals) in human.
4.     For the analysis of co-expression networks, the parameter settings should be ex-plain clearly. The parameter "r < 0.5" is arbitrary. In addition, the parameter set-ting should be provided clearly when CircAltas performs circRNA-miRNA bind-ing predictions based on miRanda, RNAhybird, and Pita.
5.     The authors should clearly describe the criteria about how to determine RBP binding peaks. In addition, the authors should provide the information/citation of the tools used to perform GO and KEGG annotations.
6.     Line 24, Page 8: "a clear tendency that MCS was negatively correlated to the tis-sue specificity index"-- Please perform a correlation test and provide the corre-sponding P value.
7.     For the portal,
(1)     the species, tissues, and sample ID (MCS-detail) should be provided (not only the number of species, tissues, and sample), when browsing a circAltas ID.
(2)     for Genome Browser, it would be better to show the coordinates of sequence fragments that form the circle sequence of the circRNA in the host gene.
(3)     Is it reasonable that the full-lengths of some circRNAs are very short (e.g., only1 bp for has-AKAP13_000126)?

Minor points:
1.     There are seven species examined in the CircAltas website, but only six species were mentioned in the text.
2.     The full list of the 1,070 RNA-seq dataset with the origin and collection of the samples should be provided. The information would be helpful for the readers.
3.     Line 2, Page 9: "This type of circRNAs was present as they are possibly generat-ed by the binding of RBPs rather than by the matching of flanking repetitive se-quences in introns." Please provide the citations.
4.     Please describe clearly the definitions of #cirexon (Fig. 4) and tissue specify score (TIS)?
5.     Line 1, Page 5: Please check whether the word "indecently" is suitable here.

**Reviewer 2**

General：
The authors build a database (CircAltas) to explore the full-length annotation and the functions of circRNA. Due to the critical roles that circRNAs played in human diseases, sequences in full-length and functional annotations of circRNAs are quite important to the RNA community. This manuscript, provided a catalog of conservative de novo annotated circRNAs from 1,070 transcriptomes of vertebrates, may be a useful resource for uncovering the hidden treasures in circRNAs. The authors did a great job in annotating circRNAs, but there were still some questions should be addressed before the acceptance of the manuscript.

Major comments:
1.     This manuscript analyzed 1,070 transcriptomes, which may have different data attributes like sequencing depths, library constructions, reads lengths, batch effects and sequencing biases, how did authors do the quality control of these sequencing data? This is very import for downstream analysis.
2.     What aligners and what parameters were used to map sequencing reads to the genome? This step is quite important for circRNA identified programs. The authors should describe them in the manuscript.
3.     It is a good idea to calculate and assign a conservation scores for circRNAs. But the so-called Multiple Conservation Score, adopted by authors, wasn't a good way to evaluate the conservation across species. They should take more factors into considerations, such as sequence similarities, BSJ locations in transcripts, conserved motifs around BSJ, number of exons involved in back-splicing, and so on. I know considering all of these will greatly complicate the computational processes, but at least some of them should be included in the conservation analysis.
4.     In line 26 on page 6, how could ChIP-seq datasets be used for identify circRNA-RBP interactions?
5.     There are too many grammatical mistakes and improper statements in the manuscript.
6.     Considering the database and the manuscript including the circRNA-RBP interactions, I suggested that the authors should describe and cite the databases related with circRNA-RBP interactions in the "Background" section, such as starBase (Li et al. Nucleic Acids Res. 2014;42:D92-D97.) and CircInteractome (RNA Biology, 2016;13(1):34-42).
7.     
To the database contents:
In detail page (circ_detail.php):
1.     There is no explanation about "Full length type".
2.     The BSJs should be labeled in different colors in circRNA sequences.
3.     What scores are used as gene and circRNA expression level? FPKM, TPM or others?
4.     Users can't get binding sites of miRNAs in "miRNA Binding" section.
5.     There is no explanation of table headers in "RBP Binding" section.

In Function analysis page:
1.     When running IRES in example data, the database prompted an error message from datatables.

In conservation analysis page:
1.     I got an error message:
Traceback (most recent call last): File "./analysis/analysis_ortholo.py", line 118, in spe_ciri2 = results1.get() File "/histor/zhao/wuwanying/anaconda2/lib/python2.7/multiprocessing/pool.py", line 572, in get raise self._value IndexError: list index out of range /usr/local/src/tomcat6/webapps/new/ortholo_analysis.php:93: Warning: /usr/local/src/tomcat6/webapps/new/tmp/ortholo/analysis/1574665677/tmp1_tmp2.bsj cannot be read [fopen] Unable to open file!

In RBP analysis page:
1.     Why would transcription factors be involved in the analysis?

**Reviewer 3**

The manuscript on CircAltas by Wanying Wu, Peifeng Ji and Fangqing Zhao is a very
important contribution to the field of circular RNA research, which suffers from
a vast sea of algorithms and databases.
A rigorous and comprehensive compilation of circRNAs is truly needed.

However, I have a few specific issues that I would like to see addressed before publication:

0) Has this database been published previously (an earlier version) ? If so, what features have been newly introduced.

1) I did not have access to any supplementary materials, whether by absence or accident, I dont know.
A minimal requirement for me would be a list of all RNA-seq libraries (protocol) used with accession numbers from NCBI SRA or a similar resource.
A potential caveat is the heterogeneity of the data : SE 50bp will perform worse than 100bp PE - Please explain which biases you would expect.

2) A major issue of existing circular RNA databases is the use of ambiguous nomenclature.
Thus, redundant or even conflicting names for circRNAs across different databases contribute to a reproducibility crisis.
How did you address this issue ? and what nomenclature do you propose ?

3) The set of genome assemblies in use is outdated.
hg19 has been replaced by hg38 in Dec 2013. Please update to hg38

4) The use of BLAT might be suboptimal for the detection of conserved BSJ.
What parameter settings were used ? USCS GB and EnsEMBL provide a vast set of whole genome alignments. Why did you decide against using them ?

5) The part on CircRNA functional annotation deserves some more attention. The value of this section is completely unclear to me.
How can CHiP-seq data help to study RNA-RBP binding ?

6) It is not clear to me how the MCS score is computed.
I understand the number of species, but tissues and individuals ?
e.g Are we talking about max, union, intersect for tissues

7) Figure 4E: Are only fully reconstituted circles included here ?
I am surprised to see that no circle is bigger than 500nt. This is probably an artifact.

**Authors’ response to reviewers**

Reviewer reports:
Reviewer #1: Wu, Ji, and Zhao developed a comprehensive database, CircAltas2.0, based on 1,070 RNA-seq samples from 19 normal tissues across six species. The authors claimed that this database contains 1,014,931 highly confident circRNAs and >80% are assembled into full-length sequences. This database is an extended version of its previous one (CircAltas1.0). The new version provides in-depth information such as conservation and functional annotation. The database is well organized and user-friendly. However, there are some concerns listed below.
Responses: We greatly appreciate the reviewer’s comments on the novelty and significance of our approach. In this revised version, we extensively revised the manuscript and added more analyses. Please refer to the following responses for detail.

Major Points:
1. Four circRNA detection tools (CIRI2, find_circ, CIRCexploer2, and DCC) were used in this database. There are dozens of publicly available circRNA detectors. The reasons why the authors selected these four tools should be provided.
Responses: Among the dozens of publicly available circRNA detection tools, CIRI2, CIRCexploer2, and DCC exhibit much better performance and lower false positive rate demonstrated by several recent benchmarking studies (Zeng et al. PLoS Comput Biol 2017, 13: e1005420; Hansen TB, Front Cell Dev Biol, 2018, 6:20). Find-circ was one of the first pipelines available to identify circRNAs from RNA-seq data and was widely used for circRNA detection. According to previous study (Hansen TB, Front Cell Dev Biol, 2018), CIRI2 combined with find_circ profited the most from combination with almost any other algorithm. Following CIRI2, we performed the downstream analysis using CIRI-AS and CIRI-full, which all based on the detected circRNAs by CIRI2. We have summarized recent progresses on circRNA detection in a review paper (Gao & Zhao, Trends in Genetics, 2018, 34: 389-400).

2. The authors claimed that the identified circRNAs were "highly confident". The authors should provide lines of evidence to support their claim (i.e., "highly confident" circRNAs). For example, do they use some strategies to further remove false positives that were derived from ambiguous alignments?
Response: For circRNA detection, four different tools were used separately on all data sets, including CIRI2, find_circ, CIRCexplorer2 and DCC. To avoid false positives, circRNAs detected by at least two tools and supported by at least two independent BSJ reads were kept as ‘highly confident’ circRNAs. This strategy has been validated by Thomas B. Hansen (Front Cell Dev Biol, 2018) and several other studies (reviewed in Kristensen et al. Nature Reviews in Genetics, 2019). These studies have shown that circRNAs filtered by combing two algorithms were highly confident and exhibited high frequency of overlap. Moreover, this strategy has been widely employed by other databases, such as circRic (Ruan et al, Genome Med, 2019, 11:55).

3. The definition of multiple conservation score (MCS) is arbitrary. How did the authors define the weighted coefficients for Ns, Nt, and Ni? Undoubtedly, ac-cording to their definition, the cis-backsplicing junctions detected from human would have the larger MCS scores as compared with those detected from other species because of much more available samples (tissues/individuals) in human.
Response: We appreciate the reviewer for pointing this out. Here, we have re-defined the MCS, which eliminates the impact of tissue or individual number on the conservation score calculation. Specifically, the newly designed measurement for each circRNA is calculated by the following formula: MCS=N_s+N_t*N_i. Briefly, for a given circRNA in a species, this formula contains two parts of number: the integer part and the decimal part. The integer part, representing the conservation across species, is the number of orthologs of this circRNA (NS). The decimal part, N_t×N_i, representing the conservation across tissues and individuals, is calculated by constructing a hierarchical expression tree (Figure 3B), in which the expression pattern of the circRNA across tissues and individuals in a species is represented by a hierarchical tree structure with two layers. The nodes in the tissue and individual layer represent the tissues and individuals in this species, respectively. Then, the score of this part is calculated by N_t×N_i, where N_t is calculated by (the number of nodes expressing circRNA in tissue layer)/(the total number of tissues), and N_s is calculated by (the summed number of nodes expressing circRNA in both layers)/2*the total number of individuals in tissues expressing circRNA. Please see Figure 3B for detail.

4. For the analysis of co-expression networks, the parameter settings should be ex-plain clearly. The parameter "r < 0.5" is arbitrary. In addition, the parameter set-ting should be provided clearly when CircAltas performs circRNA-miRNA binding predictions based on miRanda, RNAhybird, and Pita.
Response: Thank you for the suggestion. We have clarified the parameter setting in the revised manuscript (Please see Supplementary Table S3 for detail). With regard to the parameter of correlation, we have updated the networks where the correlation between nodes were initially unfiltered. Users can set the correlation cutoff by themselves when using circAtlas.

5. The authors should clearly describe the criteria about how to determine RBP binding peaks. In addition, the authors should provide the information/citation of the tools used to perform GO and KEGG annotations.
Response: Thank you for the suggestion. The RBP binding peaks used in this study was downloaded from POSTAR2 and starBase databases. The criteria for calling binding sites wad added to the manuscript (Supplementary Table S3). We have used clusterProfiler for functional enrichment. The parameters and citations have been added.

6. Line 24, Page 8: "a clear tendency that MCS was negatively correlated to the tis-sue specificity index"-- Please perform a correlation test and provide the corresponding P value.
Response: We have re-defined the MCS, which eliminates the impact of tissue or individual number. To validate the performance of this measurement, we investigated the MCS distribution of circRNAs in each species. As a result, we observed a clear tendency that MCS was positively correlated to the conservation of circRNAs in species, tissue and individual levels (P<0.001). The same results were also observed by other randomly selected windows. These results together showed that the MCS provides an effective measurement for circRNA conservation.

7. For the portal,
(1) the species, tissues, and sample ID (MCS-detail) should be provided (not only the number of species, tissues, and sample), when browsing a circAltas ID.
Response: The above information has been added.

(2) for Genome Browser, it would be better to show the coordinates of sequence fragments that form the circle sequence of the circRNA in the host gene.
Response: The coordinates are now available in our web portal.

(3) Is it reasonable that the full-lengths of some circRNAs are very short (e.g., only1 bp for has-AKAP13_000126)?
Responses: Thanks for pointing this out. We have thoroughly checked the detected circRNAs by sequence length and expression to avoid such mistake.

Minor points:
1. There are seven species examined in the CircAltas website, but only six species were mentioned in the text.
Responses: The RNA datasets of the dog species were obtained from our collaborator, which have not been published yet. We will release all the dog’s circRNA data in circAtlas soon.

2. The full list of the 1,070 RNA-seq dataset swith the origin and collection of the samples should be provided. The information would be helpful for the readers.
Responses: As suggested, we provided the detail information about RNA-seq datasets in Supplementary Table S1.

3. Line 2, Page 9: "This type of circRNAs was present as they are possibly generated by the binding of RBPs rather than by the matching of flanking repetitive sequences in introns." Please provide the citations.
Responses: As suggested, three relevant papers have been cited (Conn et al, Cell, 2015, 160:1125-1134; Ji et al. Cell Reports, 2019; Wilusz, WIRE RNA, 2020).

4. Please describe clearly the definitions of #cirexon (Fig. 4) and tissue specify score (TIS)? 5. Line 1, Page 5: Please check whether the word "indecently" is suitable here.
Responses: Cirexon is defined as the exons involved in the formation of circRNAs (Gao et al, Nature Communications, 2016, 7:12060).
We evaluated the tissue expression specificity of a given mRNA or circRNA using a previously proposed method, which varies between 0 to 1: ∑_(i=1)^n▒〖(1-expi/expmax)/(n-1)〗, where n is the number of tissues, Expi is the expression value in tissue i, and Expmax is the maximum expression level across all tissues. These definitions have been added to the manuscript.
We have carefully revised the manuscript and incorrect words have been removed.

Reviewer #2:
General：The authors build a database (CircAltas) to explore the full-length annotation and the functions of circRNA. Due to the critical roles that circRNAs played in human diseases, sequences in full-length and functional annotations of circRNAs are quite important to the RNA community. This manuscript, provided a catalog of conservative de novo annotated circRNAs from 1,070 transcriptomes of vertebrates, may be a useful resource for uncovering the hidden treasures in circRNAs. The authors did a great job in annotating circRNAs, but there were still some questions should be addressed before the acceptance of the manuscript.
Response: We greatly appreciate the valuable suggestions from the reviewer. In this revised manuscript, similar figures were combined and sequencing data sets had been released. Please refer to the following responses for detail.

Major comments:
1. This manuscript analyzed 1,070 transcriptomes, which may have different data attributes like sequencing depths, library constructions, reads lengths, batch effects and sequencing biases, how did authors do the quality control of these sequencing data? This is very import for downstream analysis.
Responses: We first performed quality control for each dataset, including removing low quality bases, filtering reads with length shorter than 50 bp and discarding data sets with low mapping quality. We detected circRNAs based on these resulting quality-controlled datasets. Expression profiles were corrected using Combat, which removes the batch effect among different data sets. The tools and parameters used in this study were described in Supplementary Table S3.

2. What aligners and what parameters were used to map sequencing reads to the genome? This step is quite important for circRNA identified programs. The authors should describe them in the manuscript.
Response: We have used four circRNA detection tools, each of which has its own recommended aligner. Specifically, BWA-mem, Tophat, STAR and Bowtie2 are used by CIRI2, find_circ, CIRCexplorer2 and DCC, respectively. As suggested, all the tools and parameters used in this study are now listed in Supplementary Table S3.

3. It is a good idea to calculate and assign a conservation score for circRNAs. But the so-called Multiple Conservation Score, adopted by authors, wasn't a good way to evaluate the conservation across species. They should take more factors into considerations, such as sequence similarities, BSJ locations in transcripts, conserved motifs around BSJ, number of exons involved in back-splicing, and so on. I know considering all of these will greatly complicate the computational processes, but at least some of them should be included in the conservation analysis.
Response: Thank you for the suggestion. In our manuscript (Figure 3A), we have described the workflow of orthologous circRNAs detection. In this pipeline, the sequence similarities, BSJ locations in transcripts, conserved motifs around BSJ and number of exons have been already considered in the conserved circRNA detection across species. We also re-designed the MCS, please refer to the third question of reviewer 1.

4. In line 26 on page 6, how could ChIP-seq datasets be used for identify circRNA-RBP interactions?
Response: We performed RBP-circRNA interaction based on CLIP-seq binding peaks downloaded from POSTAR2 and starBase. The ChIP-seq datasets were used for detecting TF binding sites. We have revised the sentences in the manuscript.

5. There are too many grammatical mistakes and improper statements in the manuscript.
Response: We have used the language editing service provided by ELSEVIER to improve our manuscript.

6. Considering the database and the manuscript including the circRNA-RBP interactions, I suggested that the authors should describe and cite the databases related with circRNA-RBP interactions in the "Background" section, such as starBase (Li et al. Nucleic Acids Res. 2014;42:D92-D97.) and CircInteractome (RNA Biology, 2016;13(1):34-42).
Response: Thanks for pointing this out. The information mentioned above has been added.

7. To the database contents:
In detail page (circ_detail.php):
1. There is no explanation about "Full length type".
Response: The explanation has been added.

2. The BSJs should be labeled in different colors in circRNA sequences.
Response: It has been revised accordingly.

3. What scores are used as gene and circRNA expression level? FPKM, TPM or others?
Response: We used FPKM for expression calculation.

4. Users can't get binding sites of miRNAs in "miRNA Binding" section.
Response: It has been revised accordingly.

5. There is no explanation of table headers in "RBP Binding" section.
Response: The explanation has been added.

In Function analysis page:
1. When running IRES in example data, the database prompted an error message from datatables.
Response: This issue has been resolved.

In conservation analysis page:
1. I got an error message:
Traceback (most recent call last): File "./analysis/analysis_ortholo.py", line 118, in spe_ciri2 = results1.get() File
"/histor/zhao/wuwanying/anaconda2/lib/python2.7/multiprocessing/pool.py", line 572, in get raise self._value IndexError: list index out of range /usr/local/src/tomcat6/webapps/new/ortholo_analysis.php:93: Warning:
/usr/local/src/tomcat6/webapps/new/tmp/ortholo/analysis/1574665677/tmp1_tmp2.bsj cannot be read [fopen] Unable to open file!
Response: This error was caused by the ‘I/O’ error of our computational cluster, where the disk was full. We have allocated more disk space for our web server.

In RBP analysis page:
1. Why would transcription factors be involved in the analysis?
Response: We have separated the TF binding analysis from RBP analysis page. Thanks for pointing this out. It has been revised accordingly.

Reviewer #3: The manuscript on CircAltas by Wanying Wu, Peifeng Ji and Fangqing Zhao is a very important contribution to the field of circular RNA research, which suffers from a vast sea of algorithms and databases. A rigorous and comprehensive compilation of circRNAs is truly needed.
Response: We greatly appreciate the reviewer’s comments on our approach. In this revised manuscript, we included more comparison suggested by the reviewer. Please refer to the following responses for detail.

However, I have a few specific issues that I would like to see addressed before publication:

0) Has this database been published previously (an earlier version)? If so, what features have been newly introduced.
Responses: In our previous study, we investigated the expression landscape and conservation of circRNAs across human, macaque and mouse and utilized mRNA-circRNA co-expression networks to infer circRNA functions and to prioritize promising functional candidates. After the publication of that paper (Ji et al. Cell Reports, 2019), a lot of requests from worldwide were sent me to ask for the access the identified circRNAs and their functional annotations. Although we have submitted the RNA-seq datasets to public databases and provided a simple web portal for the identified circRNAs (http://zhaolab.biols.ac.cn/), it's still not easier for users to access, browse, annotate and prioritize these circRNAs. Therefore, we further extended our previous study to explore circRNAs in 6 vertebrate species by integrating our own and public RNA-seq datasets and various functional annotation resources. We designed a user-friendly web portal from the scratch (http://circatlas.biols.ac.cn/).
In addition, we proposed a novel conservation scoring scheme to evaluate the conservation of circRNAs, which takes into account the species-, tissues- and individuals-level of conservation. We believe that this study, which provides a foundation for circRNA studies and serves as a powerful starting point to investigate their biological significance, would be of great interest to readers of Genome Biology.
The comparison of this study and our previous Cell Reports study is shown in Supplementary Table S2.


1) I did not have access to any supplementary materials, whether by absence or accident, I don’t know.
A minimal requirement for me would be a list of all RNA-seq libraries (protocol) used with accession numbers from NCBI SRA or a similar resource.
A potential caveat is the heterogeneity of the data: SE 50bp will perform worse than 100bp PE. Please explain which biases you would expect.
Responses: As suggested, we provided the detail information about RNA-seq datasets in Table S1. We agree with the reviewer that the heterogeneity of the public datasets may cause some biases in circRNA expression analyses. For example, identified circRNAs from RNA-seq datasets with SE-50bp should be much fewer than those from PE-150bp datasets, which may result in the underestimation of conserved circRNAs in Rat (most of them are in short sequencing length). However, with more and more sequencing data available, we will update circAtlas regularly.

2) A major issue of existing circular RNA databases is the use of ambiguous nomenclature. Thus, redundant or even conflicting names for circRNAs across different databases contribute to a reproducibility crisis. How did you address this issue? and what nomenclature do you propose?
Response: We agree with the reviewer on the nomenclature problem in this field. Hence, we designed the ‘convert’ module in our database to allow id conversion between different databases. User can input circRNA ids from any nomenclature, and the ‘convert’ module in our database will list the matched circRNA ids from other circRNA databases.

3) The set of genome assemblies in use is outdated.
hg19 has been replaced by hg38 in Dec 2013. Please update to hg38
Response: Thanks for pointing this out. We have updated the results using hg38.

4) The use of BLAT might be suboptimal for the detection of conserved BSJ.
What parameter settings were used? USCS GB and EnsEMBL provide a vast set of whole genome alignments. Why did you decide against using them?
Response: We used BLAT for conserved BSJ detection based on the reason that BLAT is more accurate and sensitive on BSJ boundary detection than the method simply based on whole genome alignment (WGA). For example, we detected conserved circRNAs between mouse and rat by using BLAT and WGA-based method, respectively. As a result, BLAT found 25,434 conserved circRNAs, whereas the WGA-based method detected 20,219 conserved circRNAs, of which more than 95% were included by the BLAT method.
To further improve its comprehensiveness and accuracy, we integrated BLAT and the WGA-based method to identify conserved circRNAs. Briefly, pairwise orthologous gene list was downloaded from the OMA orthologs database and circRNAs located on these orthologous genes for each species were determined using the genome annotation file. Subsequently, only orthologous gene pairs in which both members can express circRNAs were retained for conserved circRNA detection. Next, the boundary of the cirexon pairs in the ortholgous gene pairs were firstly determined based on the whole genome alignments which were downloaded from UCSC and Ensembl. To further confirm the conservation of these cirexons, 50-bp fragments on both sides of the circRNA BSJ were extracted and used to represent the BSJ sequence. All circRNA BSJ sequences in one species were aligned to those of the other species using BLAT, followed by the reciprocal best hits strategy to find orthologous circRNAs. Subsequently, the MultiMSOAR2.0 software, which uses a combinational approach to construct ortholog group, was employed to integrate the reciprocal best pairwise orthologous circRNAs.

5) The part on CircRNA functional annotation deserves some more attention. The value of this section is completely unclear to me. How can CHiP-seq data help to study RNA-RBP binding?
Response: We performed RBP-circRNA interaction based on CLIP-seq binding peaks downloaded from POSTAR2 and starBase. The ChIP-seq datasets were used for detecting TF binding sites. We have revised the sentences in the manuscript.

6) It is not clear to me how the MCS score is computed.
I understand the number of species, but tissues and individuals?
e.g Are we talking about max, union, intersect for tissues
Response: Species represented the conservation across species. Tissues and samples represent the expressed number of tissue and sample in each species.

7) Figure 4E: Are only fully reconstituted circles included here?
I am surprised to see that no circle is bigger than 500nt. This is probably an artifact.
Response: Yes, all the detected circRNAs were included and there are a small proportion of circRNAs with length longer than 500nt. According to our previous study, circRNA length was highly conserved among different species, with the majority of circRNAs ranging from 250 to 500 nt in length (Zheng et al. Genome Medicine, 2019, 11:2).

**Second round of review**

**Reviewer 1**

CircAltas is a useful resource, which can contribute to circRNA studies in diseases and evolutionary transcriptomics. The authors have answered most of the concerns. There are some suggestions listed below.

1.     Responses 1 and 2: To increase the reliability of the identified circRNAs, the authors can use downstream filter to further detect potential false positives from ambiguous alignments. As mentioned in Response 1, the benchmarking study (PLoS Comput Biol 2017, 13: e1005420) has provided valuable comparisons among circRNA-detection tools. A following study (PLoS Comput Biol 2019, 15: e1006158) provided a useful toolkit to detect circRNA candidates derived from ambiguous alignments. The authors can easily apply the toolkit to their circRNA candidates and add a column to indicate whether the candidates are derived from ambiguous alignments (i.e., with an alternative co-linear explanation or multiple hits).
2.     Response 3: The authors re-defined MCS as Ns + Nt*Ni. It would be better to provide an example to help users to understand the calculation of the MCS value.
3.     Response 6: To analyze the MCS values in evolution, the authors can examine the relationships between the MCS values and evolutionary rates/conservation scores (e.g., PhyloP and phastCons scores). Figs. 3C and 3D only represented the results of the mathematical formula of MCS.
4.     Response 7: The detailed list of the species, tissue, and sample name/ID should be provided when browsing a circRNA event.
5.     For the portal: When running "Conserved circRNAs", an error message popped out as follows.
"/usr/local/src/tomcat6/webapps/new/ortholo_analysis.php:93: Warning: /usr/local/src/tomcat6/webapps/new/tmp/ortholo/analysis/1580891773/tmp1_tmp2.bsj cannot be read [fopen] Unable to open file!
human     macaca"

**Reviewer 2**

The authors have answered my questions accordingly, the manuscript could be accepted.

**Reviewer 3**

None

**Authors’ response to reviewers**

Reviewer #1: CircAltas is a useful resource, which can contribute to circRNA studies in diseases and evolutionary transcriptomics. The authors have answered most of the concerns. There are some suggestions listed below.

1. Responses 1 and 2: To increase the reliability of the identified circRNAs, the authors can use downstream filter to further detect potential false positives from ambiguous alignments. As mentioned in Response 1, the benchmarking study (PLoS Comput Biol 2017, 13: e1005420) has provided valuable comparisons among circRNA-detection tools. A following study (PLoS Comput Biol 2019, 15: e1006158) provided a useful toolkit to detect circRNA candidates derived from ambiguous alignments. The authors can easily apply the toolkit to their circRNA candidates and add a column to indicate whether the candidates are derived from ambiguous alignments (i.e., with an alternative co-linear explanation or multiple hits).
Responses: We thank the reviewer for this kind suggestion. Actually, we’ve already integrated the filtration step by removing the potential false positive circRNAs derived from ambiguous alignments. As suggested, we further tried the recommended shell scripts, but failed multiple times for unknown reasons. They may be not suitable for such a huge amount of data. Both references have been cited and discussed in this revised manuscript.

2. Response 3: The authors re-defined MCS as Ns + Nt*Ni. It would be better to provide an example to help users to understand the calculation of the MCS value.
Responses: As suggested, we provided an example on how to calculate the MCS value (Supplementary Figure S1).

3. Response 6: To analyze the MCS values in evolution, the authors can examine the relationships between the MCS values and evolutionary rates/conservation scores (e.g., PhyloP and phastCons scores). Figs. 3C and 3D only represented the results of the mathematical formula of MCS.
Responses: As suggested, we calculated the correlation between MCS scores and phyloP/phastCons scores (Supplementary Figure S2).

4. Response 7: The detailed list of the species, tissue, and sample name/ID should be provided when browsing a circRNA event.
Responses: Thanks. It has been revised accordingly.

5. For the portal: When running “Conserved circRNAs”, an error message popped out as follows. “/usr/local/src/tomcat6/webapps/new/ortholo_analysis.php:93: Warning: /usr/local/src/tomcat6/webapps/new/tmp/ortholo/analysis/1580891773/tmp1_tmp2.bsj cannot be read [fopen] Unable to open file!
human macaca"
Response: Thanks for pointing this out. It has been revised accordingly.

Reviewer #2: The authors have answered my questions accordingly, the manuscript could be accepted.

Reviewer #3: I deem this manuscript acceptable for publication
